# Transition in sexual system and sex chromosome evolution in the tadpole shrimp *Triops cancriformis*

**DOI:** 10.1038/hdy.2015.10

**Published:** 2015-03-11

**Authors:** T C Mathers, R L Hammond, R A Jenner, B Hänfling, J Atkins, A Gómez

**Affiliations:** 1 School of Biological, Biomedical and Environmental Sciences, University of Hull, Hull, UK; 2 Department of Biology, University of Leicester, Leicester, UK; 3 Department of Life Sciences, The Natural History Museum, London, UK

## Abstract

Transitions in sexual system and reproductive mode may affect the course of sex chromosome evolution, for instance by altering the strength of sexually antagonistic selection. However, there have been few studies of sex chromosomes in systems where such transitions have been documented. The European tadpole shrimp, *Triops cancriformis*, has undergone a transition from dioecy to androdioecy (a sexual system where hermaphrodites and males coexist), offering an excellent opportunity to test the impact of this transition on the evolution of sex chromosomes. To identify sex-linked markers, to understand mechanisms of sex determination and to investigate differences between sexual systems, we carried out a genome-wide association study using restriction site-associated DNA sequencing (RAD-seq) of 47 males, females and hermaphrodites from one dioecious and one androdioecious population. We analysed 22.9 Gb of paired-end sequences and identified and scored >3000 high coverage novel genomic RAD markers. Presence–absence of markers, single-nucleotide polymorphism association and read depth identified 52 candidate sex-linked markers. We show that sex is genetically determined in *T. cancriformis*, with a ZW system conserved across dioecious and androdioecious populations and that hermaphrodites have likely evolved from females. We also show that the structure of the sex chromosomes differs strikingly, with a larger sex-linked region in the dioecious population compared with the androdioecious population.

## Introduction

Sex chromosomes have evolved independently from autosomes numerous times in plants and animals and represent an extraordinary case of evolutionary convergence with shared features indicating the action of similar evolutionary forces (Fridolfsson *et al.*, 1998; Lahn and Page, 1999; Skaletsky *et al.*, 2003; Graves, 2008; Pease and Hahn, 2012). The established model of sex chromosome evolution states that genetic sex determination by a dominant allele coupled with sexually antagonistic selection can lead to the evolution of non-recombining, morphologically differentiated sex chromosomes (Charlesworth *et al.*, 2005; Bergero and Charlesworth, 2009; Bachtrog *et al.*, 2011). Sexually antagonistic selection, where alleles may benefit one sex and harm the other, is widespread in groups with genetically determined sexes (for example, Rice, 1992; Foerster *et al.*, 2007; Cox and Calsbeek, 2009) and is a critical creative force in the evolution of morphologically differentiated sex chromosomes (Charlesworth and Charlesworth, 1980; Rice, 1987a; van Doorn and Kirkpatrick, 2010; Jordan and Charlesworth, 2012; Qiu *et al.*, 2013; Charlesworth *et al.*, 2014; Kirkpatrick and Guerrero, 2014; Otto, 2014). Transitions in sexual system and reproductive mode can alter the strength of sexually antagonistic selection, and so may potentially affect the course of sex chromosome evolution. Studying the sex chromosomes of species that have undergone such transitions in sexual system is therefore important, however, species with labile sexual systems have been little studied (Pires**-**daSilva, 2007; Bachtrog *et al.*, 2014).

Tadpole shrimps (Crustacea: Notostraca), although renowned for their morphological stability (Fryer, 1988; Mathers *et al.*, 2013a), show diverse sexual systems including dioecy (gonochorism), hermaphroditism and androdioecy (AD; self-fertile hermaphrodites and males) (Sassaman, 1991). AD is a sexual system where hermaphrodites and a small proportion of males coexist within populations (Weeks *et al.*, 2006a). Phylogenetic character mapping of sexual systems has shown that AD is likely to have evolved from dioecy independently multiple times (Mathers *et al.*, 2013b). The European tadpole shrimp, *Triops cancriformis*, has undergone one such transition. In this species, populations in Spain are dioecious and obligate outcrossers, whereas in the rest of Europe there are either obligatory selfing hermaphroditic populations or androdioecious populations with hermaphrodites and a reduced proportion of males, where both selfing and outcrossing occurs (Sassaman, 1991; Zierold *et al.*, 2007, 2009). Importantly, phylogeographic analysis has revealed that hermaphroditism (hermaphrodite and androdioecious) has evolved from dioecy (Zierold *et al.*, 2007; Mathers *et al.*, 2013b).

This transition makes *T. cancriformis* potentially an illuminating model, but, currently there is no information on sex determination. A ZW genetic sex determination system (with heterogametic females) is the most likely model as in two other androdioecious branchiopod species, *Eulimnadia texana* and *Triops newberryi,* maleness is recessive to hermaphroditism (Sassaman, 1989, 1991; Sassaman and Weeks, 1993; Otto *et al.*, 1993). However, karyological analysis identified 2*n*=12 chromosomes, with no morphologically distinct sex chromosomes in *T. cancriformis* (Ombretta *et al.*, 2005). In addition, selfing experiments on *E. texana* and *T. newberryi* indicate two types of hermaphrodites in androdioecious populations: amphigenics (ZW), which produce males and hermaphrodites in a 1:3 ratio, and monogenics (WW), which produce only hermaphrodites (Sassaman, 1991; Sassaman and Weeks, 1993).

Investigating biologically interesting questions in non-model species with few genomic resources has been greatly aided by developments in next-generation sequencing. Restriction site-associated DNA sequencing (RAD-seq) is a genotyping-by-sequencing approach that has proved particularly useful (Davey and Blaxter, 2010; Reitzel *et al.*, 2013). RAD-seq can generate thousands of single-nucleotide polymorphism (SNP) markers in tens to hundreds of individuals (Baird *et al.*, 2008; Hohenlohe *et al.*, 2010), and it has been successfully used to identify sex-linked markers (Anderson *et al.*, 2012; Carmichael *et al.*, 2013; Palaiokostas *et al.*, 2013; Gamble and Zarkower, 2014).

Here, using a genome-wide association approach, we explicitly test a ZW model of genetic sex determination in *T. cancriformis* and investigate the effect of a transition in sexual system on patterns of sex chromosome evolution. We used RAD-seq to score thousands of genetic RAD markers (‘markers') in males and females from a dioecious population and males and hermaphrodites from an androdioecious population of *Triops cancriformis* to identity sex-linked markers. We show that sex is genetically determined in *T. cancriformis*, with a ZW system conserved across dioecious and androdioecious populations, and a smaller sex-specific region in the androdioecious population.

## Materials and methods

### Samples, rearing and individual sexing

Sediment samples containing *T. cancriformis* diapausing cysts were obtained from a dioecious population in Espolla (ESP), Spain, and an androdioecious population in pond 12 in Königswartha (KOE), Germany (Zierold *et al.*, 2007, 2009). Cysts were hatched by mixing sediment with purified water in small tanks kept at 20 °C under 24-h illumination, with resulting hatchlings reared on *Triops* food (www.triops.es). Mature individuals were fixed in 100% ethanol and sexed before genetic analysis with individuals without ovisacs considered male and those with ovisacs females (ESP) or hermaphrodites (KOE) (Mathers *et al.*, 2013b).

### Sample preparation and DNA extraction

Pre-extraction, the digestive tract of each individual was removed from the abdominal section and the gut cavity flushed with 100% ethanol to minimise contamination with exogenous DNA. Genomic DNA was extracted from ~1-cm long abdominal sections using the DNAeasy Blood and Tissue Kit (Qiagen, Venlo, Netherlands) including an RNase A digestion step. DNA quality was assessed on 0.8% agarose gels and quantified by fluorimetry (Quant-iT PicoGreen Kit, Invitrogen, Paisley, UK). In total, 14 males and 12 females from the dioecious population (ESP) and 6 males and 15 hermaphrodites from the androdioecious population (KOE), each yielding at least 18 ng μl^−1^ of high molecular weight DNA, were used in the RAD libraries.

### RAD library preparation and sequencing

Four paired-end RAD libraries were prepared by the NERC/NBAF facility at the University of Edinburgh (The GenePool) following Baird *et al.* (2008) with some modifications (Ogden *et al.*, 2013). For each individual, 1 μg total genomic DNA was digested using the restriction enzyme *SbfI* followed by ligation of a barcoded P1 RAD adapter (for details, see Supplementary Table S1). Samples were then split into four multiplexed libraries before sonic shearing, size selection (300–700 bp) and ligation of P2 RAD adapters. Libraries were PCR amplified, quantified and sequenced in separate flow cells on an Illumina (San Diego, CA, USA) HiSeq 2000 platform with 100 bp, paired-end chemistry (v1 chemistry). Two sequencing runs were carried out and the raw reads concatenated *in silico* for each library.

### Sequence quality control and trimming

**Figure 1:**
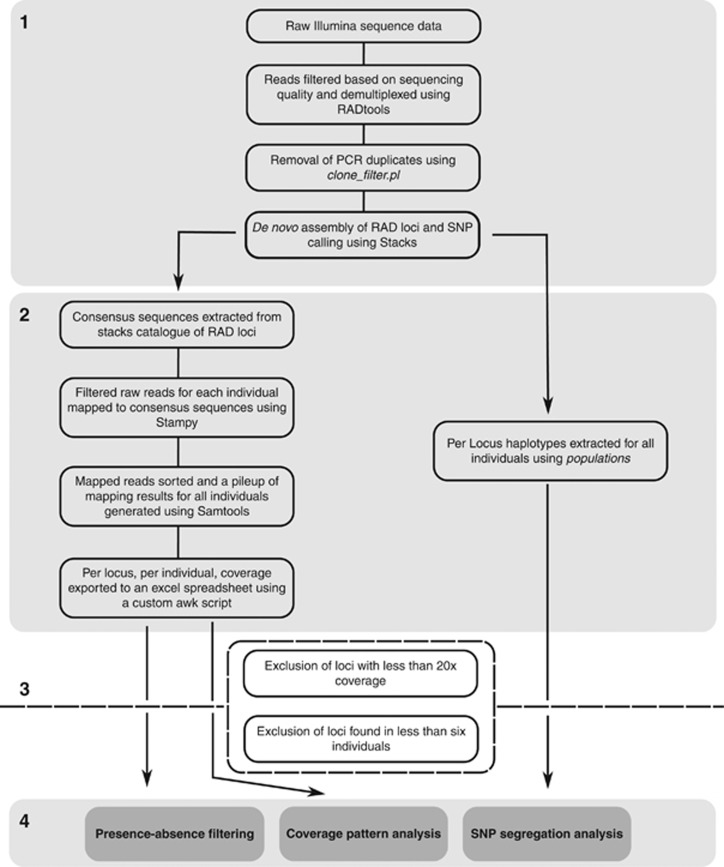
Pipeline used to process the RAD-seq data.

The pipeline used to process and analyse RAD-seq data is summarised in Figure 1. Sequencing quality of each RAD library was assessed with FastQC (http://www.bioinformatics.bbsrc.ac.uk/projects/fastqc). We then used RADtools (Baxter *et al.*, 2011) to trim reads to 70 bp and remove sequences lacking a correct barcode or *Sbf*I restriction site, or with quality score <20 at any position. To account for sequencing errors, a fuzzy matching algorithm was used allowing 1- bp deviation from the expected restriction site and barcode sequences (Baxter *et al.*, 2011). First- and second-end reads were then de-multiplexed based on their barcode sequence.

### *De novo* assembly of RAD markers and SNP calling

Genotyping-by-sequencing necessitates the grouping of sequences into homologous loci and allelic scoring at these loci. RAD-seq is a reduced representation method that generates sequences at either side of a restriction site (Baird *et al.*, 2008), however, as there is no genomic reference for *Triops cancriformis* to map reads to, it was impossible to make this association. We therefore considered sequences with sufficient similarity (six mismatches or less) to be homologous, and we defined these groups of similar sequences as RAD markers. Variants within each RAD marker were defined as RAD alleles. We avoided the term ‘locus' as our RAD-seq approach provided a mixture of both presence–absence markers, likely caused by the presence–absence of a restriction site, and also allelic variation.

Assembly of RAD markers and SNP calling was performed using components of Stacks v. 0.99993 (Catchen *et al.*, 2011). PCR duplicates were removed using clone_filter.pl. First-end reads for each individual were then clustered into candidate RAD markers using ustacks. At least 15 identical reads per individual were required to call an allele and up to six mismatches allowed between alleles of the same marker (we allowed six mismatches because our focus was the identification of sex-linked, and therefore potentially divergent, markers). SNPs were simultaneously called using the built in maximum likelihood diploid genotyping model (Hohenlohe *et al.*, 2010). Candidate RAD markers with coverage two standard deviations above the average were removed as they likely were repetitive elements (Catchen *et al.*, 2011, 2013). A catalogue of all RAD markers found across the sampled individuals was constructed using cstacks with markers considered to be homologous if they had six or fewer mismatches between individuals. Then, sstacks was used to identify genotypes for each RAD marker for every individual and the populations programme used to export genotypes for each marker of every individual and to calculate population summary statistics and population differentiation (*F*_ST_).

To obtain coverage for each candidate RAD marker per individual, raw first-end reads from each individual were mapped back to consensus sequences from every RAD marker in the Stacks catalogue using Stampy (Lunter and Goodson, 2011) with default settings. SAMtools (Li *et al.*, 2009) was then used to generate a pileup from which coverage information (per marker, per individual) was extracted.

Using this approach, we created a ‘filtered catalogue' containing high coverage RAD markers (>20 reads) that were found in six or more individuals (the number of males in KOE, the smallest gender sample) to be used in all downstream analyses. This filtered catalogue was used to test the expectations of marker presence–absence, RAD marker coverage and SNP segregation, predicted by a ZW sex determination model. As total per individual coverage varies in RAD libraries (Richards *et al.*, 2013; Wagner *et al.*, 2013), we normalised per marker coverage for each individual by dividing by the median coverage of the subset of RAD markers in the catalogue that were found in all 47 individuals. These were consider to be a large sample (800 markers, see Results section) of candidate autosomal markers.

### Testing predictions for ZW chromosomal sex determination

Candidate completely sex-linked markers were first identified in females and males from ESP (dioecious) and hermaphrodites and males from KOE (androdioecious) based on patterns predicted by an explicit ZW model (Table 1). We used presence–absence of RAD markers, together with marker coverage, to identify markers specific to particular sex chromosomes (W- or Z-specific markers). We also used the distribution of alleles between the sexes to identify markers present on both sex chromosomes but with SNP alleles associated with a particular sex chromosome (sex-linked alleles).

In addition, we compared the numbers of RAD markers in linkage disequilibrium (LD) with the completely sex-linked makers identified above between the dioecious and androdioecious populations. Genepop files containing genotypes for all markers from the filtered catalogue found in all individuals for each population were generated using populations (Catchen *et al.*, 2011). Genepop On The Web version 4.2 (http://genepop.curtin.edu.au/) (Raymond and Rousset, 1995) was used to test for LD using default settings. We first investigated patterns of pairwise LD in both populations and then looked for pairs of markers in significant LD that involved at least one of our completely sex-linked markers.

## Results

### Sequencing

A total of 114 304 783 read pairs were obtained from the four RAD libraries comprising 22.9 Gb of sequence (Supplementary Table S2). After trimming, quality control and PCR duplicate removal we retained 28 986 269 70-bp read pairs (4.06 Gb). Individual coverage ranged from 246 930 (KOE_12_H2) to 1 390 021 reads (ESP_M11) with an average of 616 729 reads per sample (*s*=242 257) (Supplementary Table S3).

### Assembly of RAD markers, SNP calling and population genetic diversity and differentiation

Stacks *de novo* assembled 20 902 candidate RAD markers. After filtering for RAD markers with >20 reads found in six or more individuals, the filtered catalogue contained 3822 markers, which accounted for 93% of the approximately 26 million retained reads that were mappable to the catalogue. Of these, 800 RAD markers were found in all individuals and had consistently high coverage (median=299x; see Supplementary Table S3). Based on 1559 markers found in all individuals of at least one of the two populations (which excludes sex chromosome-specific markers) *F*_ST_ between ESP and KOE was 0.71, in close agreement to microsatellite-based estimates between the same populations (Zierold *et al.*, 2009) and confirming substantial population genetic differentiation. The level of within population polymorphism varied with 28.9% (351/1221) polymorphic markers (containing at least one SNP) in ESP and just 9.5% (122/1288) in KOE.

### Dioecious and androdioecious populations of *T. cancriformis* have ZW chromosomal sex determination

**Figure 2:**
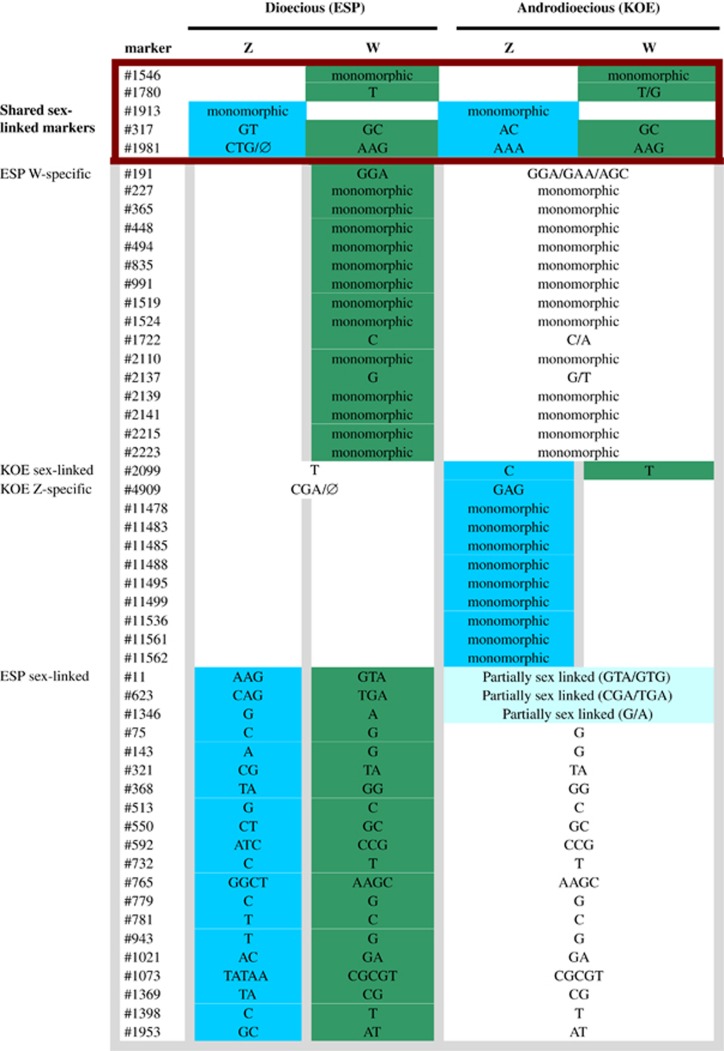
Summary of the candidate sex-linked RAD markers found in the dioecious (ESP) and androdioecious (KOE) populations. We note that this is an overview of the patterns of sex linkage found in our data set, not a genetic map. We show both markers specific for a particular sex chromosome (W and Z specific) and also markers with complete sex linkage (sex-linked markers). SNPs present in polymorphic RAD markers are indicated in the corresponding cells. Strong colours indicate complete sex linkage (blue for Z linked and green for W linked), whereas paler colours represents markers that are in LD to the sex-linked markers in the population-level LD analysis. Sex-linked markers shared between ESP and KOE are enclosed in a red box.

**Figure 3:**
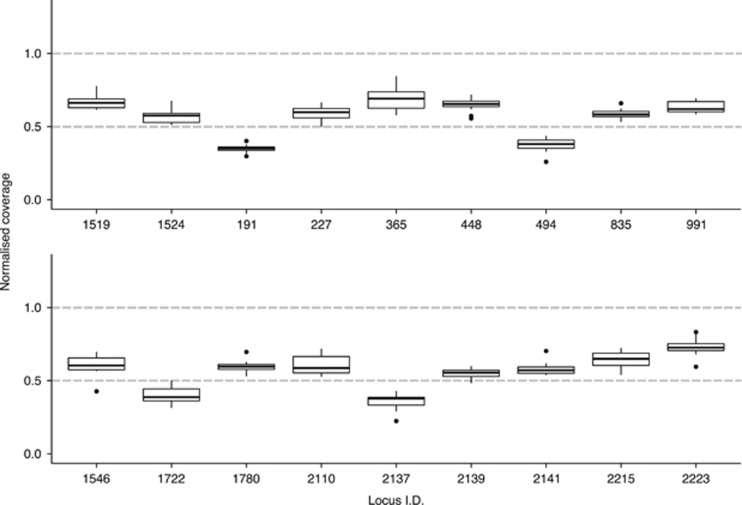
Normalised coverage of 18 candidate W-specific markers in ESP females. Dotted lines show the expected coverage for autosomal markers (1.0), and hemizygous markers (0.5). All markers were identified based on patterns of presence–absence.

We searched the 3822 markers in the filtered catalogue according to our predictions for a ZW chromosomal sex determination system (Table 1) to identify completely sex-linked markers in the dioecious (ESP) and androdioecious (KOE) populations. In the dioecious population (ESP), 18 RAD markers met the criteria for putative W-specific markers (Figure 2): they were present in all ESP females (*n*=12, putatively ZW) and absent in all ESP males (*n*=14, putatively ZZ), had coverage consistent with being hemizygous (Figure 3) and all females had a single allele. Analysis of ESP genotype data recovered an additional 21 completely sex-linked RAD markers with alleles segregating in a manner consistent with a ZW chromosomal sex determination system, where females were always heterozygous and had female-specific (W-linked) alleles and the males always homozygous with Z-linked alleles (Figure 2). In contrast, our data were incompatible with a heterogametic XY male model because no markers were present in all males (Y specific) and absent in all females, nor were any markers found with sex-linked alleles where males were always heterozygous (Y linked).

**Figure 4:**
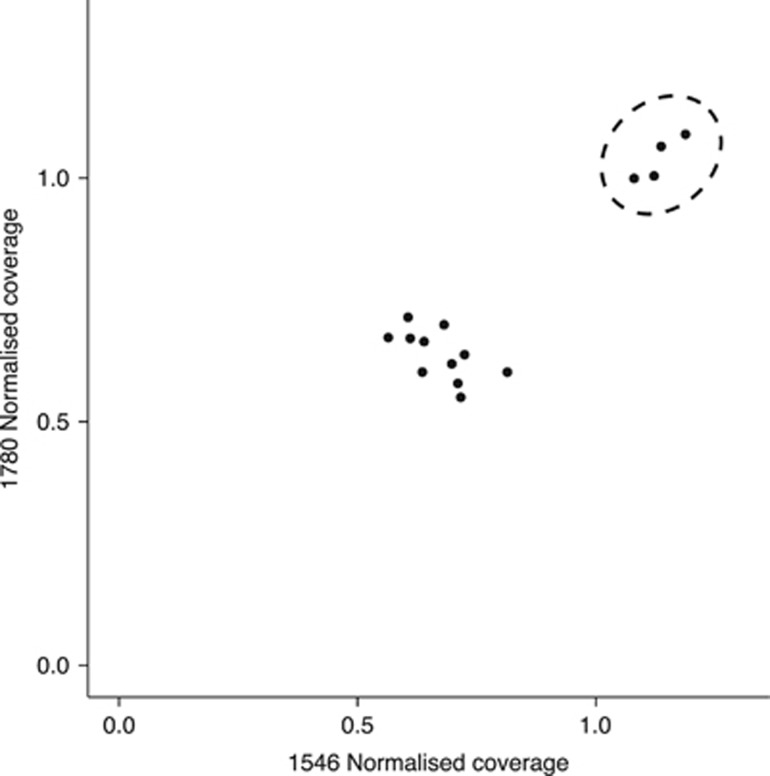
Identification of KOE monogenic (WW) and amphigenic (WZ) hermaphrodites. Normalised coverage of both W-specific markers (#1780 and #1546) found in KOE for 15 KOE hermaphrodites. The two clusters show amphigenic (ZW) hermaphrodites with coverage approximately half that of autosomal markers (as expected under hemizygosity) and monogenic (WW) hermaphrodites (dashed circle) with coverage equivalent to that of autosomal markers. Four monogenic (WW) hermaphrodites were identified: 1: KOE_12_H29, 2: KOE_12_H25, 3: KOE_12_H10, 4: KOE_12_H21.

Just two markers, also previously identified by filtering in ESP, were putatively W specific (#1546 and #1780; see Figure 2) in the androdioecious population (KOE), being found in all hermaphrodites (*n*=15, putatively WW or ZW) but absent in all males (*n*=6, putative ZZ). Using these markers, we were able to test another expectation of a ZW chromosomal sex determinations system in an androdioecious population—the presence of monogenic (WW) and amphigenic (ZW) hermaphrodites. W-specific markers are expected to have approximately equivalent coverage to that of autosomal markers in monogenics (WW) and half that of autosomal markers in amphigenics (ZW) (Table 1). Plotting normalised coverage for the two W-specific markers against each other for all KOE hermaphrodites revealed a cluster of four putative monogenic (WW) hermaphrodites with coverage of around 1 for both markers and a second cluster of 11 putative amphigenic (WZ) hermaphrodites with coverage of around 0.5 for both markers (Figure 4).

**Figure 5:**
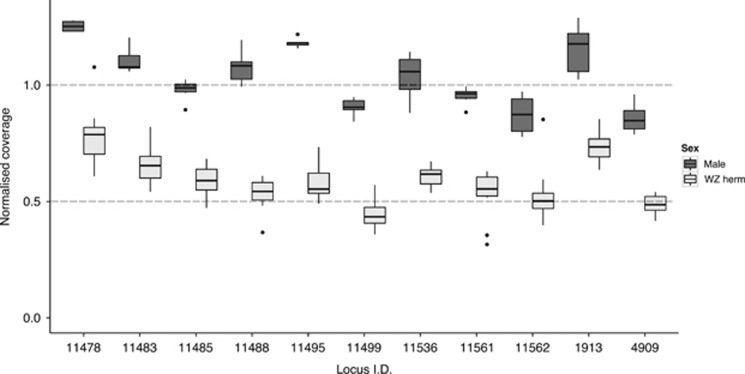
Male (ZZ, dark grey) vs amphigenic hermaphrodite (ZW, pale grey) normalised coverage for 11 candidate Z-specific markers in KOE. Dotted lines show the expected coverage for autosomal markers (1.0), and hemizygous markers (0.5). These markers were absent in monogenic (WW) KOE hermaphrodites.

Identification of Z-specific markers in KOE was facilitated by the identification of monogenic hermaphrodites (WW). Filtering the catalogue for markers present in all males (*n*=6, putatively ZZ) and all amphigenic hermaphrodites (*n*=11, putatively ZW) but absent in all monogenic hermaphrodites (*n*=4, putatively WW) recovered 11 candidate Z-specific markers. As expected, these markers had ~0.5 normalised coverage in amphigenic ZW hermaphrodites compared with normalised coverage of ~1 in ZZ males, further supporting the Z-specific status of these markers (Figure 5). Just 2 of the 11 candidate Z-specific markers in KOE were found in ESP. One of them, #1913, showed coverage expected for a Z-specific marker (0.5 normalised coverage in females, ZW, and 1 in males, ZZ), but patterns of presence–absence in the other marker, #4909, were incompatible with complete sex linkage (data not shown). In the dioecious population (ESP), patterns of presence–absence are uninformative to identify additional Z-specific markers as both males (ZZ) and females (ZW) are expected to carry at least one Z-linked allele.

Analysis of KOE genotype data revealed a further three completely sex-linked markers (#317, #1981 and #2099) with alleles segregating as expected for a ZW chromosomal sex determination system. These markers were heterozygous in all amphigenic hermaphrodites (*n*=11, putatively ZW) and homozygous in all monogenic hermaphrodites (*n*=4, putatively WW) and males (*n*=6, putatively ZZ), and all markers had hermaphrodite-specific (W-linked) alleles. All three sex-linked markers were also present in ESP, one was monomorphic (#2099), another was also sex-linked (#317) and the third one (#1981) had polymorphism and coverage patterns consistent with being completely sex linked, but with a null allele segregating in the Z chromosome, so it was not identified following our specific criteria in Table 1 (data not shown), so it was added to our list of sex-linked markers.

Overall, we identified 52 putative completely sex-linked markers across both populations (Figure 2) and confirmed ZW genetic sex determination in *T. cancriformis*, with two types of hermaphrodite in the androdioecious population (monogenics and amphigenics). Five markers are completely sex linked in both populations, suggesting conservation of a core sex-determining region. Of these, two markers are W specific (#1546 and #1780), one Z specific (#1913) and two have completely sex-linked alleles (#317 and #1981) with the phase preserved in both populations (Figure 2). Coverage across RAD markers identified as having completely sex-linked alleles was consistently high in all individuals with median coverage across markers ranging from 133 to 412 × .

### The transition between dioecy and AD is associated with altered patterns of sex linkage

Although both dioecious (ESP) and androdioecious (KOE) populations show patterns of marker segregation consistent with ZW chromosomal sex determination, the size of the sex-linked region is smaller in the androdioecious population. All 41 completely sex-linked markers (Figure 2) identified in the dioecious population (ESP) are recovered in KOE but only five remain completely sex linked. Furthermore, only 12 additional completely sex-linked markers are found in KOE even including Z-specific markers, which cannot be reliably identified in ESP (Figure 2). The remaining 37 sex-linked markers in ESP were also found in KOE, but the sex-linked status could not be ascertained in 30 of them, which were monomorphic with the ESP W-specific allele and present in all individuals (Figure 2). The three polymorphic markers were in significant LD with sex-linked markers in the population analysis in KOE (markers #11, #1346 and #623; Figure 2, see below); two have the ESP W allele along with additional alleles not found in ESP; and the other has both the ESP W and Z allele (see Figure 2).

**Figure 6:**
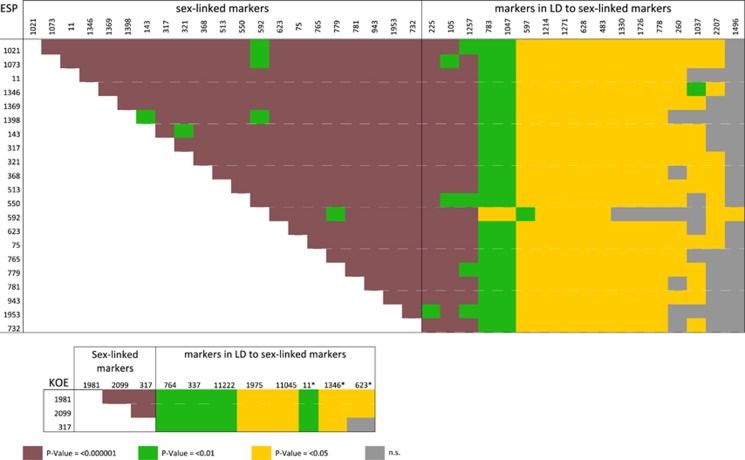
Population linkage analysis. RAD markers in LD with identified sex-linked markers are shown for each population. Asterisks indicate markers that are sex linked in the ESP population.

We investigated patterns of population sex linkage using tests of LD. In ESP, where, compared with KOE, there were a greater number of polymorphic markers (351 vs 122), just 5% of pairs of markers (2871 out of 58 311) were significantly in LD. In contrast, in KOE, 19% of pairs of markers (1204/6328) were significantly in LD, indicating higher levels of genome-wide population LD relative to ESP. We then looked for marker pairs in significant LD where one marker was known from our previous filtering to be sex linked. In ESP, 17% (497 out of 2871) involved at least one sex-linked marker, with 17 markers significantly linked to most of the 21 completely sex-linked markers, three of them highly significantly (#225, #105 and #1257). In KOE, in contrast, although a higher percentage of markers were in LD genome wide, just 2% of pairs of markers (24 out of 1204) included the three sex-linked markers. These involved a total of eight markers, three of them completely sex-linked in ESP (#11, #1346 and #623), which were significantly linked to the three completely sex-linked markers (Figure 6).

We note that our criteria to discover completely sex-linked markers (Table 1) excluded sex-linked markers where polymorphism occurs *within* either sex chromosome (including null alleles, linked to restriction site polymorphism). However, this is likely to involve a small number of markers and will not affect our conclusions. Polymorphic markers within sex chromosomes, if they occur, should appear in LD to the completely sex-linked markers in the population. Therefore, we checked the haplotypes of all markers in LD with sex-linked markers in ESP and KOE in the population-level linkage analysis and did not find any that were polymorphic within either sex chromosome and completely sex linked. However, we identified one putative sex-linked marker (#1981; Figure 2), shared between populations, which had null alleles segregating in the Z chromosome.

## Discussion

Our analysis confirmed chromosomal sex determination in *T. cancriformis*. Specifically, our prediction of a ZW sex determination system was upheld in both populations, with males from the dioecious and androdioecious populations homogametic (ZZ), females from the dioecious population heterogametic (ZW) and two types of hermaphrodites in the androdioecious populations (monogenic WW and amphigenic ZW). The percentage of monogenics (27%) is similar to that found in *Eulimnadia* (Weeks *et al.*, 1999, 2014). Our data also showed that both *T. cancriformis* populations share a small set of completely sex-linked markers, suggesting conservation of a core sex-determining region, presumably containing the master sex determination gene. Finding ZW sex determination adds support to this sex determination mechanism being broadly conserved in Branchiopoda, as it is found not only in the androdioecious American species *T. newberryi*, where maleness is determined by a recessive locus (Sassaman 1991), but also in the more distantly related clam shrimp *Eulimnadia texana* (Sassaman and Weeks, 1993; Weeks *et al.*, 2010) and the brine shrimp *Artemia* (Stefani, 1963; Legrand *et al.*, 1987; Parraguez *et al.*, 2009; De Vos *et al.*, 2013).

### Structure of *Triops cancriformis* sex chromosomes

Our results suggest striking differences between the structure of sex chromosomes in the androdioecious (KOE) and dioecious (ESP) populations, with fewer sex-linked markers in the androdioecious population, suggesting a smaller sex-specific region. We also found decreased differentiation between the putative W and Z chromosomes, as the Z chromosome in KOE contains a region almost identical to the W. In addition, we found a greater proportion of marker pairs in significant LD that contained a completely sex-linked marker (‘sex-linked pairs') in the dioecious (ESP) compared with the androdioecious (KOE) population. This was despite the greater proportion of marker pairs in LD over the whole genome in KOE compared with ESP. The higher LD seen over the whole genome in KOE is likely a direct effect of increased selfing by hermaphrodites (Nordborg, 2000) and a correlated reduction in effective population size. However, we also observed that a much higher proportion of sex-linked pairs were in LD compared with genome-wide pairs in ESP. Such a contrast suggests that the dioecious population (ESP) has a larger sex-specific, non-recombining region, despite lower levels of genome-wide LD.

The sizes of the sex-linked regions in both populations can be roughly estimated assuming that sex-linked markers are physically linked. Our RAD library was made using the restriction enzyme *SbfI*, which has an 8-bp recognition site. Given that *Triops* mean GC content is 0.47 (estimated from the RAD autosomal markers), this results in two RAD markers flanking the cut site approximately every 100 kb (see ‘RAD counter' on https://www.wiki.ed.ac.uk/display/RADSequencing/Home). As there are 41 completely sex-linked markers in the dioecious population (corresponding to *ca* 20 restriction sites), these sex-linked markers are expected to cover *ca* 2 Mb of W-linked sequence. In contrast, the W-specific region in the androdioecious population is much smaller, with just 3 W-linked RAD sites corresponding to around 300 kb. However, these estimated sizes should be interpreted with caution, given, that *SbfI* is expected to target GC-rich regions, and we do not know if there are any GC biases in the sex-linked regions.

### Evolution of sex chromosomes

The strength of sexually antagonistic selection in the dioecious population is expected to be much higher than in the AD population. Given our ZW genetic model, the W is always found in females (ZW) in dioecious populations, so selection for female-benefitting mutations and recombination suppression between these markers and the sex-determining region is likely as is the case for male-benefitting mutations on the Y chromosome (Charlesworth and Charlesworth, 1980; Rice, 1987a; Bergero and Charlesworth, 2009; Kirkpatrick and Guerrero, 2014). The Z, meanwhile, would be subjected to mild male-biased sexually antagonistic selection as, assuming a 50:50 sex ratio, the Z is found 2/3 of the time in males and 1/3 in females. In contrast, in androdioecious populations, both the W and the Z are found mostly in hermaphrodites, which must balance both male and female function. The W is exclusively found in hermaphrodites (in both WW monogenics and ZW amphigenics), but the Z is largely found in hermaphrodites too because males (ZZ) are rare and the Z is also carried by amphigenic (ZW) hermaphrodites. Although some sexually antagonistic selection is expected to occur in hermaphroditic plants and animals (Bedhomme *et al.*, 2009; Abbott, 2011), it seems highly likely that sexually antagonistic selection is a stronger force in dioecious compared with androdioecious populations. The smaller sex-linked region and the lower proportion of markers in LD to the sex-linked region in KOE compared with ESP (Figure 6) is broadly compatible with the hypothesis that recombination suppression is relaxed in KOE compared with ESP, a possibility that requires more research. The production of hermaphroditic individuals (effectively, putatively functional intersex individuals) in *Eulimnadia* has been attributed to a low level of recombination between sex chromosomes (Weeks *et al.*, 2006b). However, and given that in many organisms, recombination depends on phenotypic and not genotypic sex (Kawamura and Nishioka, 1977; Matsuba *et al.*, 2010), there is also the possibility that increased recombination could be brought about by the presence of Z and W chromosomes in the testis lobes of ZW hermaphrodites, which undergo spermatogenesis. In a ZW system with males and females, recombination is expected to be higher during spermatogenesis in ZZ males (the homogametic sex) than during oogenesis, so a transition to hermaphroditism may be directly followed by increased ZW recombination (Perrin, 2009; Guerrero *et al.*, 2012). However, the patterns of variation between the Z and W in KOE, with the KOE Z having high similarity to the ESP W, suggest that differences in recombination patterns is not the complete story.

Surprisingly, most completely sex-linked markers found in ESP are also found in KOE, but in KOE they are fixed for the ESP W haplotype. This suggests there is a large region where the KOE Z and W chromosomes are very similar, with both strongly resembling the haplotype now found in ESP W. This is, in principle, surprising, as the W in ESP is non-recombining in the heterogametic sex, so under traditional models it would be expected to accumulate deleterious mutations that would be compensated by the Z in the heterogametic sex in ESP (Rice, 1987b, 1994; Bachtrog, 2006). The similarity of W and Z in KOE to the ESP W is therefore paradoxical as there would be no compensation.

A second surprising finding is that, in contrast to the pattern found in W chromosomes, the Z chromosomes from ESP and KOE show marked differentiation in the non-recombining region, with 10 Z-specific RAD markers found only in KOE. From the four shared markers with Z-specific alleles, only one of them (#1913) has the same haplotype in ESP and KOE. This is notwithstanding the large region now shared between KOE W and Z haplotypes. This suggests that while the W chromosome-determining hermaphroditism may have originated from a dioecious population, genetically similar to ESP, one or more regions of the Z chromosome may have originated from introgression from a population of a different geographic origin. This hypothesis is supported by the presence of two divergent mitochondrial lineages in *T. cancriformis* in Central Europe, including KOE, which are likely to originate in different glacial refugia (Zierold *et al.*, 2007). Therefore, our findings are compatible with a stepwise evolution of AD, in which first, WW hermaphrodites evolved from a dioecious population related to ESP and expanded though Europe. The second step, the evolution of AD from hermaphroditism would follow. Indeed, the small geographical area where AD occurs in Europe (Zierold *et al.*, 2007) is located in a contact zone between divergent mtDNA lineages, lending strength to the hypothesis that AD may have evolved from hermaphroditism because of male invasion.

### Evolution of AD in *Triops cancriformis*

Given that the W sex chromosomes are highly similar between ESP females and KOE hermaphrodites and that the sex determination mode is conserved—with femaleness and hermaphroditism dominant over maleness—our data are consistent with hermaphrodites having evolved from females rather than males. It also agrees with the inference of a recent transition between dioecy and hermaphroditism/AD as proposed by Zierold *et al.* (2007). The evolution of hermaphrodites from females, rather than males in sexually dimorphic animals is expected to occur because of developmental constraints—it is simpler to evolve a self-fertilising hermaphrodite from a female rather than a male (Weeks *et al.*, 2006c; Weeks, 2009, 2012). In females, only a mutation causing sperm production in the ovaries, to produce an ovotestis, is required for the evolution self-fertile hermaphrodites. In contrast, the evolution of hermaphrodites from males would be more complex as males lack important female traits, such as the ovisac and nest-building behaviour, and possess unimportant behaviours, such as mate searching.

Simple ovotestis, in which sperm-producing testis lobes are scattered in the ovary are found in androdioecious and hermaphroditic species of *Triops*, including *T. cancriformis* (Longhurst, 1955; Akita, 1971; Scanabissi *et al.*, 2005; Garcia**-**Velazco *et al.*, 2009; Murugan *et al.*, 2009), suggesting this pathway to hermaphroditism could be conserved across the genus, possibly linked to a recombination event producing intersex individuals, as has been suggested for *Eulimnadia* (Weeks *et al.*, 2006a). The low number of shared sex-linked RAD markers between the two populations, and the presence of only one additional W-linked marker in KOE suggests that these markers may be tightly linked to a possibly conserved master sex-determining locus, and likely also to the gene determining hermaphroditism in androdioecious populations. Further characterisation of these regions of the sex chromosomes, and investigation of these markers in related androdioecious taxa, could shed light on the genetic changes involved in the evolution of hermaphroditism in *Triops* and whether this mechanism is indeed conserved across the genus.

## Conclusions

Evolutionary transitions from genetically determined separate sexes to AD offer a unique opportunity to investigate the evolution of sex chromosomes, including testing the role of sexually antagonistic selection in the evolution of sex-associated linkage. By *de novo* assembly of RAD markers and testing patterns of marker presence–absence, coverage, SNP association and linkage as predicted by a specific genetic model, we have uncovered the genetic mode of sex determination, identified putative sex-linked markers and established differences in the size of the sex-linked regions between populations, with lower differentiation between sex chromosomes in the androdioecious population. The presence of differentiated sex chromosomes in *T. cancriformis* alongside recently evolved diverse sexual systems (Zierold *et al.*, 2007 2009) makes the species an excellent model for the study of sex chromosome evolution. Future work should extend the study of sex chromosomes across Notostraca, where multiple transitions in sexual system have occurred (Mathers *et al.*, 2013b), to bring further insights into the genomic effects of labile sexual systems.

## Data archiving

The sequence data produced in this study are available via the Sequence Read Archive through accession number PRJEB7851 (http://www.ebi.ac.uk/ena/data/view/PRJEB7851). Additional data are included in Dryad: http://doi.org/10.5061/dryad.4635t.

## Figures and Tables

**Table 1:** Predicted segregation patterns for completely sex-linked RAD markers given a ZW chromosomal sex determination system in dioecious (ESP) and androdioecious (KOE) populations

*Population*	*ESP*		*KOE*		
*Sexual system*	*Dioecious*		*Androdioecious*		
*Sex*	*Female*	*Male*	*Hermaphrodite*		*Male*
			*Monogenic*	*Amphigenic*	
Genetic model (sex-linked allele segregation pattern)	ZW	ZZ	WW	ZW	ZZ
					
*W-specific markers*					
Pattern	+	Ø	+	+	Ø
Coverage^a^	0.5	−	1.0	0.5	−
Polymorphism	Hem.	−	Hom. or het.	Hem.	−
					
*Z-specific markers*					
Pattern	+	+	Ø	+	+
Coverage^a^	0.5	1.0	−	0.5	1.0
Polymorphism	Hem.	Hom. or het.	−	Hem.	Hom. or het.

Abbreviations: ESP, Espolla; hem., hemizygous; het., heterozygous; hom., homozygous; KOE, Königswartha; RAD, restriction site-associated DNA; SNP, single-nucleotide polymorphism.

For RAD markers with completely sex-linked alleles, SNP segregation patterns are shown. For RAD markers specific to either the Z or W, sex chromosome predicted patterns are shown for presence (+)/absence (Ø), marker coverage and zygosity (hem., hom. or het.).

a Coverage relative to autosomal markers found in all individuals in both populations.
