# Supplementary material for: Transition in sexual system and sex chromosome evolution in the tadpole shrimp Triops cancriformis
Source: Heredity (Edinb). 2015 Mar 11;115(1):37–46. doi: 10.1038/hdy.2015.10 (PMC4815504; doi:10.1038/hdy.2015.10)
Supplement: Supplementary Tables [file hdy201510x1.docx]

Table S1: Sample information for all individuals included in the four RAD libraries. In total 12 barcodes were used and split across the 4 RAD libraries.

| **Sample I.D.** | **Adapter barcode** | **RAD library I.D.** | **Collection location** | **Sex** |
| --- | --- | --- | --- | --- |
| ESP_F10 | TCAGTGCT | Lib_2 | Espolla, Spain | Female |
| ESP_F11 | TCTCGCTC | Lib_2 | Espolla, Spain | Female |
| ESP_F13 | TGACATAC | Lib_2 | Espolla, Spain | Female |
| ESP_F14 | TGTGACTG | Lib_2 | Espolla, Spain | Female |
| ESP_F15 | ACACTGAC | Lib_3 | Espolla, Spain | Female |
| ESP_F17 | ACGTCTAC | Lib_3 | Espolla, Spain | Female |
| ESP_F19 | AGAGTCGA | Lib_3 | Espolla, Spain | Female |
| ESP_F20 | TACGCTCG | Lib_2 | Espolla, Spain | Female |
| ESP_F2 | GCTACAGC | Lib_2 | Espolla, Spain | Female |
| ESP_F4 | GTCACTCA | Lib_2 | Espolla, Spain | Female |
| ESP_F5 | GTGTACTG | Lib_2 | Espolla, Spain | Female |
| ESP_F8 | TAGCGACG | Lib_2 | Espolla, Spain | Female |
| ESP_M10 | CATGTCGT | Lib_3 | Espolla, Spain | Male |
| ESP_M11 | CGATCAGC | Lib_3 | Espolla, Spain | Male |
| ESP_M1 | GATCGTGA | Lib_4 | Espolla, Spain | Male |
| ESP_M12 | CGTACTCG | Lib_3 | Espolla, Spain | Male |
| ESP_M13 | CTAGCTCT | Lib_3 | Espolla, Spain | Male |
| ESP_M14 | CTGATGCT | Lib_3 | Espolla, Spain | Male |
| ESP_M15 | GACTACGA | Lib_4 | Espolla, Spain | Male |
| ESP_M16 | GTCACTCA | Lib_4 | Espolla, Spain | Male |
| ESP_M2 | AGTCTGCT | Lib_3 | Espolla, Spain | Male |
| ESP_M3 | GCATGTGC | Lib_4 | Espolla, Spain | Male |
| ESP_M5 | GCTACAGC | Lib_4 | Espolla, Spain | Male |
| ESP_M7 | ATCGCAGC | Lib_3 | Espolla, Spain | Male |
| ESP_M8 | ATGCTGTA | Lib_3 | Espolla, Spain | Male |
| ESP_M9 | CACATGAC | Lib_3 | Espolla, Spain | Male |
| KOE_12_H10 | ACGTCTAC | Lib_1 | Pond 12, Königswartha, Germany | Hermaphrodite |
| KOE_12_H18 | AGAGTCGA | Lib_1 | Pond 12, Königswartha, Germany | Hermaphrodite |
| KOE_12_H19 | AGTCTGCT | Lib_1 | Pond 12, Königswartha, Germany | Hermaphrodite |
| KOE_12_H20 | ATCGCAGC | Lib_1 | Pond 12, Königswartha, Germany | Hermaphrodite |
| KOE_12_H21 | ATGCTGTA | Lib_1 | Pond 12, Königswartha, Germany | Hermaphrodite |
| KOE_12_H2 | ACACTGAC | Lib_1 | Pond 12, Königswartha, Germany | Hermaphrodite |
| KOE_12_H23 | CACATGAC | Lib_1 | Pond 12, Königswartha, Germany | Hermaphrodite |
| KOE_12_H24 | CATGTCGT | Lib_1 | Pond 12, Königswartha, Germany | Hermaphrodite |
| KOE_12_H25 | CGATCAGC | Lib_1 | Pond 12, Königswartha, Germany | Hermaphrodite |
| KOE_12_H26 | CGTACTCG | Lib_1 | Pond 12, Königswartha, Germany | Hermaphrodite |
| KOE_12_H27 | CTAGCTCT | Lib_1 | Pond 12, Königswartha, Germany | Hermaphrodite |
| KOE_12_H28 | GACTACGA | Lib_2 | Pond 12, Königswartha, Germany | Hermaphrodite |
| KOE_12_H29 | GATCGTGA | Lib_2 | Pond 12, Königswartha, Germany | Hermaphrodite |
| KOE_12_H30 | GCATGTGC | Lib_2 | Pond 12, Königswartha, Germany | Hermaphrodite |
| KOE_12_H4 | CTGATGCT | Lib_1 | Pond 12, Königswartha, Germany | Hermaphrodite |
| KOE_12_M10 | TGACATAC | Lib_4 | Pond 12, Königswartha, Germany | Male |
| KOE_12_M1 | GTGTACTG | Lib_4 | Pond 12, Königswartha, Germany | Male |
| KOE_12_M3 | TACGCTCG | Lib_4 | Pond 12, Königswartha, Germany | Male |
| KOE_12_M4 | TAGCGACG | Lib_4 | Pond 12, Königswartha, Germany | Male |
| KOE_12_M5 | TCAGTGCT | Lib_4 | Pond 12, Königswartha, Germany | Male |
| KOE_12_M6 | TCTCGCTC | Lib_4 | Pond 12, Königswartha, Germany | Male |

Table S2: Summary of sequencing results and quality control for the 4 RAD libraries.

| **Library** | **Number of read pairs** | **Not in RAD format** | **No Sbf1 site** | **No matching barcode** | **Quality score below 20** | **PCR duplicates*** | **Retained read pairs** |
| --- | --- | --- | --- | --- | --- | --- | --- |
| Lib_1 | 25664353 | 6831566 | 693342 | 18358 | 5374735 | 5922130 | 6824222 |
| Lib_2 | 36108312 | 9864384 | 1166594 | 9839 | 7266784 | 8714967 | 9085744 |
| Lib_3 | 23500808 | 6274717 | 808301 | 15027 | 5043105 | 4334274 | 7025384 |
| Lib_4 | 29031310 | 7796305 | 980922 | 9620 | 5865785 | 8327759 | 6050919 |
| **Total** | 114304783 | 30766972 | 3649159 | 52844 | 23550409 | 27299130 | **28986269** |
| **% Reads** |  | 26.92 | 3.19 | 0.05 | 20.60 | 23.88 | **25.36** |

* PCR duplicates refers to the number of duplicates removed from the cleaned reads, not the number of duplicates in the unprocessed data.

**Table S3:** Sequencing results for all 47 individuals included in the RAD analysis.

| **Sample I.D.** | **Library** | **Reads** | **Reads (PCR duplicates removed)** | **% PCR duplicates** | **Median coverage for autosomal loci*, n=800 (Inter quartile range)** |
| --- | --- | --- | --- | --- | --- |
| ESP_F10 | Lib_2 | 1017151 | 571231 | 56.16 | 327 (284 – 369) |
| ESP_F11 | Lib_2 | 1536925 | 1023316 | 66.58 | 307 (259 – 348) |
| ESP_F13 | Lib_2 | 999051 | 601611 | 60.22 | 278 (242 – 317) |
| ESP_F14 | Lib_2 | 825697 | 491439 | 59.52 | 260 (220 – 297) |
| ESP_F15 | Lib_3 | 619632 | 402848 | 65.01 | 239 (208 – 268) |
| ESP_F17 | Lib_3 | 856391 | 567920 | 66.32 | 286 (246 – 322) |
| ESP_F19 | Lib_3 | 575059 | 374487 | 65.12 | 232 (198 – 264) |
| ESP_F2 | Lib_2 | 1510484 | 804893 | 53.29 | 430 (369 – 493) |
| ESP_F20 | Lib_2 | 1960923 | 954832 | 48.69 | 527 (460 – 607) |
| ESP_F4 | Lib_2 | 688107 | 398325 | 57.89 | 247 (213 – 281) |
| ESP_F5 | Lib_2 | 2513056 | 1103002 | 43.89 | 640 (557 – 747) |
| ESP_F8 | Lib_2 | 2726913 | 1214613 | 44.54 | 658 (571 – 755) |
| ESP_M1 | Lib_4 | 855669 | 380230 | 44.44 | 224 (186 – 263) |
| ESP_M10 | Lib_3 | 621032 | 390134 | 62.82 | 233 (202 -258) |
| ESP_M11 | Lib_3 | 2239455 | 1390021 | 62.07 | 469 (410 – 531) |
| ESP_M12 | Lib_3 | 1042976 | 651546 | 62.47 | 322 (280 – 357) |
| ESP_M13 | Lib_3 | 1365719 | 749766 | 54.90 | 457 (362 – 521) |
| ESP_M14 | Lib_3 | 754455 | 459783 | 60.94 | 278 (246 – 310) |
| ESP_M15 | Lib_4 | 1047355 | 457514 | 43.68 | 246 (206 – 291) |
| ESP_M16 | Lib_4 | 1483267 | 611595 | 41.23 | 353 (296 – 412) |
| ESP_M2 | Lib_3 | 911196 | 561635 | 61.64 | 323 (285 – 363) |
| ESP_M3 | Lib_4 | 827404 | 396570 | 47.93 | 186 (155 – 220) |
| ESP_M5 | Lib_4 | 1054979 | 448650 | 42.53 | 281 (235 – 333) |
| ESP_M7 | Lib_3 | 835119 | 522363 | 62.55 | 308 (262 – 342) |
| ESP_M8 | Lib_3 | 941464 | 562073 | 59.70 | 346 (306 – 381) |
| ESP_M9 | Lib_3 | 597160 | 392808 | 65.78 | 204 ( 176 – 228) |
| KOE_12_H10 | Lib_1 | 928315 | 490450 | 52.83 | 277 (222 – 334) |
| KOE_12_H18 | Lib_1 | 894009 | 474034 | 53.02 | 259 (210 – 306) |
| KOE_12_H19 | Lib_1 | 1457456 | 795620 | 54.59 | 334 (276 – 411) |
| KOE_12_H2 | Lib_1 | 418394 | 246930 | 59.02 | 139 (111 – 172) |
| KOE_12_H20 | Lib_1 | 968154 | 500571 | 51.70 | 278 ( 224 – 342) |
| KOE_12_H21 | Lib_1 | 1419720 | 748391 | 52.71 | 356 (288 – 424) |
| KOE_12_H23 | Lib_1 | 1059799 | 536921 | 50.66 | 289 (235 – 348) |
| KOE_12_H24 | Lib_1 | 782487 | 404452 | 51.69 | 240 (190 – 289) |
| KOE_12_H25 | Lib_1 | 1318478 | 671620 | 50.94 | 348 (281 – 419) |
| KOE_12_H26 | Lib_1 | 970011 | 497629 | 51.30 | 286 (231 – 344) |
| KOE_12_H27 | Lib_1 | 1222727 | 704986 | 57.66 | 263 (212 – 319) |
| KOE_12_H28 | Lib_2 | 626983 | 382946 | 61.08 | 197 (171 – 224) |
| KOE_12_H29 | Lib_2 | 2501148 | 1068521 | 42.72 | 617 (542 – 699) |
| KOE_12_H30 | Lib_2 | 894273 | 471015 | 52.67 | 298 (262 – 335) |
| KOE_12_H4 | Lib_1 | 1306802 | 752618 | 57.59 | 287 (235 – 350) |
| KOE_12_M1 | Lib_4 | 1106998 | 481117 | 43.46 | 273 (231 – 317) |
| KOE_12_M10 | Lib_4 | 1337745 | 542403 | 40.55 | 335 (286 – 388) |
| KOE_12_M3 | Lib_4 | 1983935 | 789837 | 39.81 | 454 (383 – 527) |
| KOE_12_M4 | Lib_4 | 1261096 | 537857 | 42.65 | 313 (265 – 362) |
| KOE_12_M5 | Lib_4 | 1440629 | 611423 | 42.44 | 343 (292 – 399) |
| KOE_12_M6 | Lib_4 | 1979601 | 793723 | 40.10 | 456 (394 – 526) |

*Loci found in all individuals with a minimum of 20x coverage are assumed to be autosomal.
